# Unraveling the mystery of fever of unknown origin: a remarkable journey towards the diagnosis of peripheral T-cell lymphoma-T follicular helper type: A rare case report

**DOI:** 10.1097/MD.0000000000036974

**Published:** 2024-01-19

**Authors:** Petar I. Trifonov, Vesselin K. Koltchakov, Raynichka P. Mihaylova-Garnizova, Aleksandar Y. Yordanov, Liliya Sokolova, Rosen K. Nikolov, Zahariy A. Krastev

**Affiliations:** a Clinic of Gastroenterology, UMHAT St. Ivan Rilski, Sofia, Bulgaria; b Clinic of Infectious Diseases, Military Medical Academy, Sofia, Bulgaria; c Clinic of Hematology, UMHAT St. Ivan Rilski, Sofia, Bulgaria.

**Keywords:** FUO, lymphoma, PTCL-TFH lymphoma

## Abstract

**Introduction:**

Fever of unknown origin (FUO) remains one of the most challenging clinical conditions. It demands an exhaustive diagnostic approach, considering its varied etiologies spanning infectious, autoimmune, inflammatory, and malignant causes.

**Patient concerns:**

This report shows the journey of diagnosing a 28-year-old male who presented with persistent fever and lower-extremity weakness over 9 months. Despite seeking care at multiple hospitals, a definitive diagnosis remained elusive.

**Diagnosis:**

The patient underwent a series of evaluations in various specialties, including gastroenterology, infectious diseases, rheumatology, hematology, and cardiology. Multiple tests and treatments were administered, including antiviral therapy for hepatitis B and antibiotics for suspected infections.

**Interventions:**

After an initial misdiagnosis and unsuccessful treatments, a positron emission tomography-computed tomography scan and lymph node biopsy ultimately led to the diagnosis of peripheral T-cell lymphoma-T follicular helper type (PTCL-TFH) lymphoma. The patient was referred to the hematology clinic and initiated on CHOEP (cyclophosphamide, vincristine, etoposide, and prednisone) chemotherapy.

**Outcomes:**

The patient showed a positive response to CHOEP therapy, as indicated by a posttreatment positron emission tomography-computed tomography scan. He reported a significant improvement in his quality of life. Additional rounds of the same regimen were planned to further manage the lymphoma.

**Conclusion:**

This case emphasizes the importance of a comprehensive and persistent diagnostic approach in managing FUO. Initially, the focus on infectious causes led to extensive treatments, but the disease’s progression and complications shifted attention to other specialties. The eventual diagnosis of PTCL-TFH lymphoma highlights the significance of advanced imaging techniques and multidisciplinary collaboration in uncovering elusive diagnoses. Thorough surveillance, timely reassessments, and repeated testing can uncover definitive changes critical for diagnosis. PTCL-TFH lymphoma, although rare, should be considered in the differential diagnosis of FUO, especially when initial evaluations are inconclusive.

## 1. Introduction

Fever of unknown origin (FUO) is a challenging clinical problem that requires extensive diagnostic evaluation. Despite exhaustive workup, the underlying cause of FUO remains unclear in some cases. In this case report, we present a rare and challenging case of a 28-year-old patient with fever over the course of 9 months, who after nearly 60 consecutive days in 2 different clinics was diagnosed with peripheral T-cell lymphomas of the T follicular helper type (PTCL-TFH lymphoma). It is a rare subtype of non-Hodgkin lymphoma that is characterized by the proliferation of malignant T cells with TFH cell-like features.

## 2. Clinical case

A 28-year-old male patient of Romani descent was diagnosed with chronic hepatitis B for a half-year long septic condition presented at our medical facility in a wheelchair seeking medical evaluation and treatment for his liver disease. The purpose of his visit was to obtain further diagnostic clarifications and to determine the most appropriate course of treatment.

The patient described a progressive onset of lower-extremity weakness from March 2022 over a period of 7 months, along with muscle and joint pain, diminished appetite, high-grade fever (reaching 40°C), and a self-resolving maculopapular chest rash. The patient had no history of other diseases or previous illnesses, and denied any family illnesses upon admission. The patient did not consume alcohol nor smoke.

**Figure 1.**
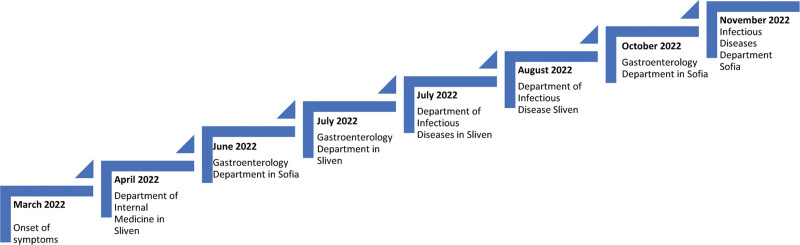
Chronological overview of the patient’s hospital admissions.

Following these symptoms, he sought diagnostic assessments and treatments at multiple hospitals (Fig. 1).

### 2.1. Internal medicine—1 time

- April 2022 in Sliven because of fever and weakness. Elevated liver enzymes were observed and the patient was referred to gastroenterology department.

### 2.2. Gastroenterology—3 times

**Figure 2.**
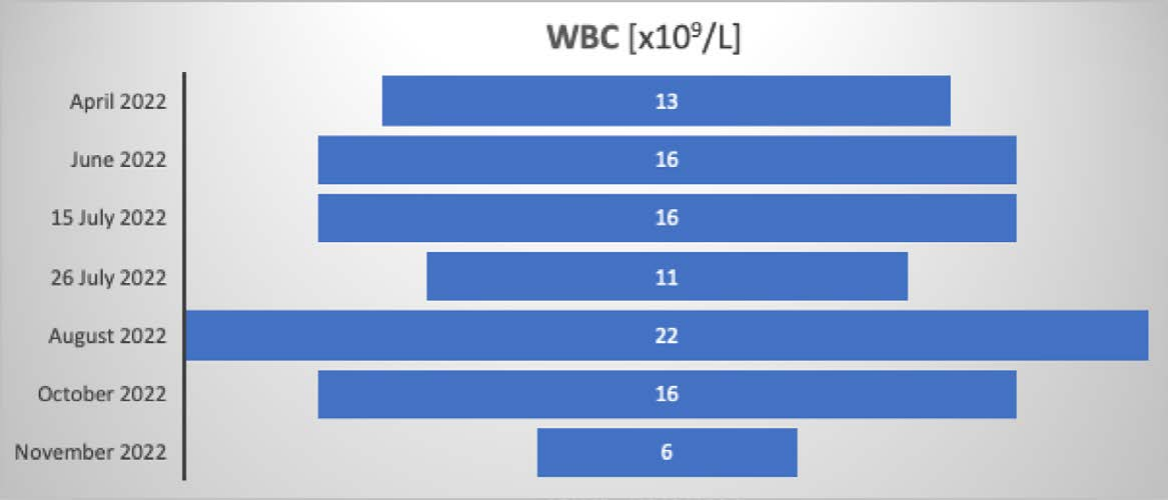
White blood cell count across multiple hospital admissions.

**Figure 3.**
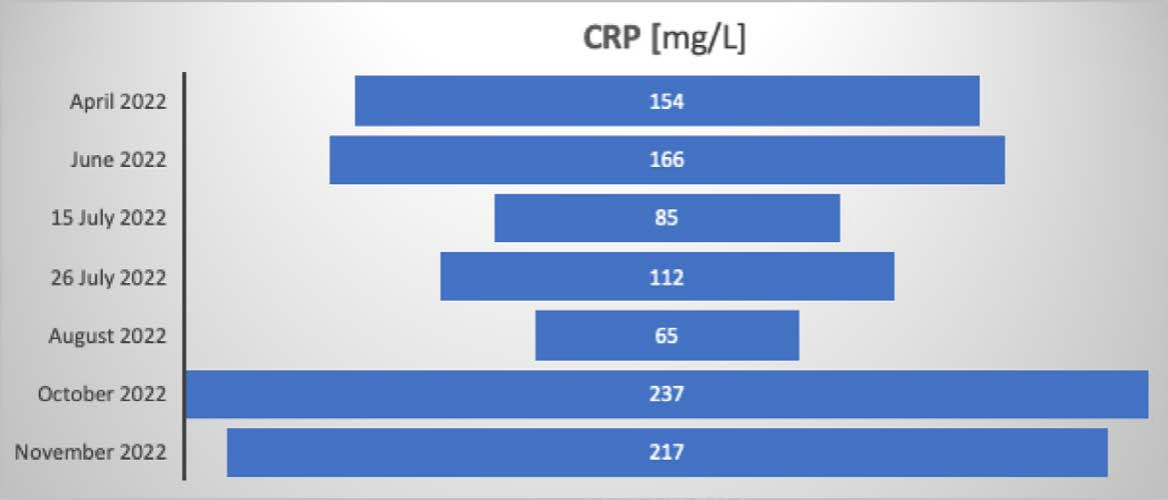
C-reactive protein (CRP) across multiple hospital admissions.

**Figure 4.**
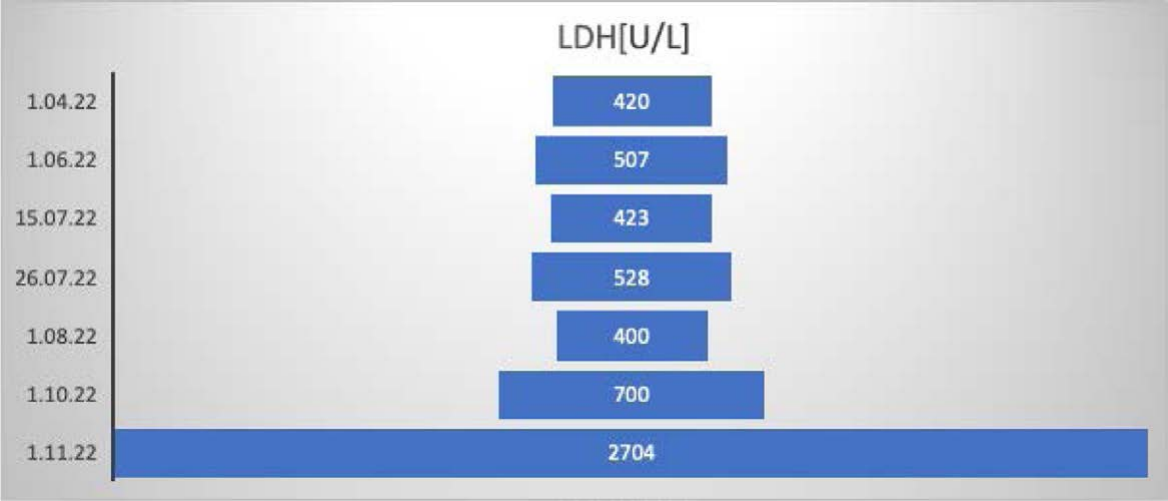
Lactate dehydrogenase (LDH) across multiple hospital admissions.

**Figure 5.**
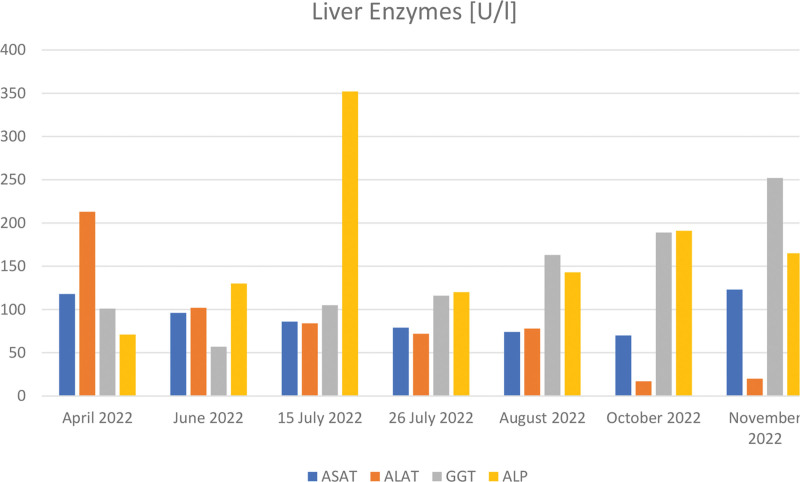
Aspartate aminotransferase (ASAT), alanine transaminase (ALAT), gamma-glutamyl transferase (GGT), and alkaline phosphatase (ALP) across multiple hospital admissions.

- June 2022 was referred to the Gastroenterology Department in Sofia due to elevated liver enzymes levels. HBV-DNA was undetectable, and anti-hepatitis D virus (HDV) antibodies was positive. Leukocytosis and anemia were also observed. All laboratory findings from April to November 2023 are shown in Figures 2–5. The data are presented as absolute values, without the ability to establish a reference range because it fluctuates between different laboratories.
- July 2022—Gastroenterology Department in Sliven, with leukocytosis and fever, and received additional antibiotic treatment. Imaging procedures revealed liver cirrhosis, enlarged liver and spleen, and ascites
- From October to November in Sofia: The patient had a viral load of 4172 IU/mL, positive anti-HDV, and nondetectable HDV RNA. Imaging procedures showed liver cirrhosis, enlarged liver and spleen, ascites, pleural effusions, portal hypertension, and esophageal varices grade 1 with portal gastropathy seen on esophagogastroduodenoscopy. Treatment with Tenofovir was initiated.

During the hospital stay an unexpected increase in cholestatic enzymes levels (gamma-glutamyl transferase and alkaline phosphatase) was noted. A second abdominal ultrasound was conducted, which revealed newly formed small liver granulomas.

Ziehl-Neelsen sputum tests were performed on 3 separate days, and a TB-Spot test was also conducted.

While the TB-Spot test results were pending, the first Ziehl-Neelsen test returned positive, indicating the presence of *Mycobacterium tuberculosis*. Consequently, a treatment regimen comprising of Azithromycin, Rifampicin, and Ethambutol was initiated. However, after 7 days without discernible improvement and a negative TB-Spot test result and the second and third Ziehl-Neelsen tests, the treatment was discontinued.

### 2.3. Infectious diseases—2 times

- First admission in Infectious Disease Department in July 2023 in Sliven. Negative test results were obtained for urine culture, sputum analysis, *Rickettsia conorii*, and Lyme IgM and IgG levels. Treatment with meropenem and doxycycline were also administered.
- In August 2022, the patient was readmitted to the Department of Infectious diseases. The patient was tested for various infections, including HIV (anti-HIV), *Coxiella burnetii* (IgM and IgG), brucellosis (*Brucella* species IgM and IgG), Leishmaniasis (*Leishmania infantum* IgM and IgG), and *Borrelia burgdorferi* (anti-*B burgdorferi* IgM and IgG). All of the test results were negative. Corticosteroids, meropenem, and levofloxacin were prescribed. The patient was afebrile for the first time in 5 months after a 10-day treatment course but experienced symptom recurrence a few days later.

### 2.4. Rheumatology—3 times

- In Sliven—antinuclear antibody screening and rheumatoid factor test and possible rheumatoid arthritis was suspected.
- October 2022 in Sofia the patient was diagnosed with adult-onset Still disease. Treatment with methylprednisolone was started but later deemed ineffective.
- Anti-Mi-2alpha, anti-Mi-2 beta, anti-TIF1 gamma, anti-MDA5, anti-NXP2, anti-SAE1, anti-Ku, anti-PM-Scl100, anti-PM-Scl75, anti-Jo1, anti-SRP, anti-PL-7, anti-PL-12, anti-EJ, anti-OJ, anti-Ro 52, C3, C4, and anti-ds DNA, all of which were within the reference range. Despite the presence of joint symptoms, rheumatic disease was conclusively excluded from the diagnosis.

### 2.5. Hematology—3 times

- April 2022—the patient’s anemia was likely attributed to chronic inflammation and hypersplenism.
- November 2022—insufficient evidence of hematological pathology, and the anemic syndrome was probably associated with the patient’s hepatic illness and possible underlying undiagnosed thalassemia. Bone marrow trephine biopsy was performed.

### 2.6. Cardiology

- Echocardiographic examination: no evidence of organic changes in the valves or vegetation; the presence of pericardial effusion was confirmed.

### 2.7. Parasitology consultations

- It was conducted following positive results for *Mycoplasma pneumoniae*. Polymerase chain reaction testing was conducted, which determined that this was a cross-reaction.

### 2.8. Infectious diseases reevaluation in Sofia

Following a 6-month period of disease assessment, a comprehensive reevaluation of potential infectious diseases was undertaken. Corticosteroids and antibiotics were discontinued. Further testing for *Mycoplasma pneumoniae* IgM, *Chlamydia pneumoniae* IgM, *Coxiella burnetii* IgG, *Trichinella* IgG, *Leishmania* IgG, and *Toxocara* IgG was performed, yielding negative results. On the seventh day after medication cessation, a follow-up CT scan was performed, revealing axillary lymphadenopathy, pericardial and pleural effusions, hepatosplenomegaly, and abdominal lymphadenomegaly.

The results from the trephine biopsy were obtained 14 days after the procedure was performed. The immunomorphological findings do not allow for an independent diagnostic evaluation and should be interpreted within the broader clinical-laboratory context. In differential diagnosis—reactive changes in the inflammatory process/manifestation of a chronic myeloproliferative or myelodysplastic process.

Subsequently, a positron emission tomography-computed tomography (PET-CT) scan was performed, followed by an axillary lymph node biopsy. Ten months post the initial onset of symptoms, the final diagnosis was reached: PTCL-TFH lymphoma. Upon establishing the diagnosis, the patient was directed to the Hematology Clinic, where a regimen of CHOEP (cyclophosphamide, vincristine, etoposide, and prednisone) treatment was initiated.

**Figure 6.**
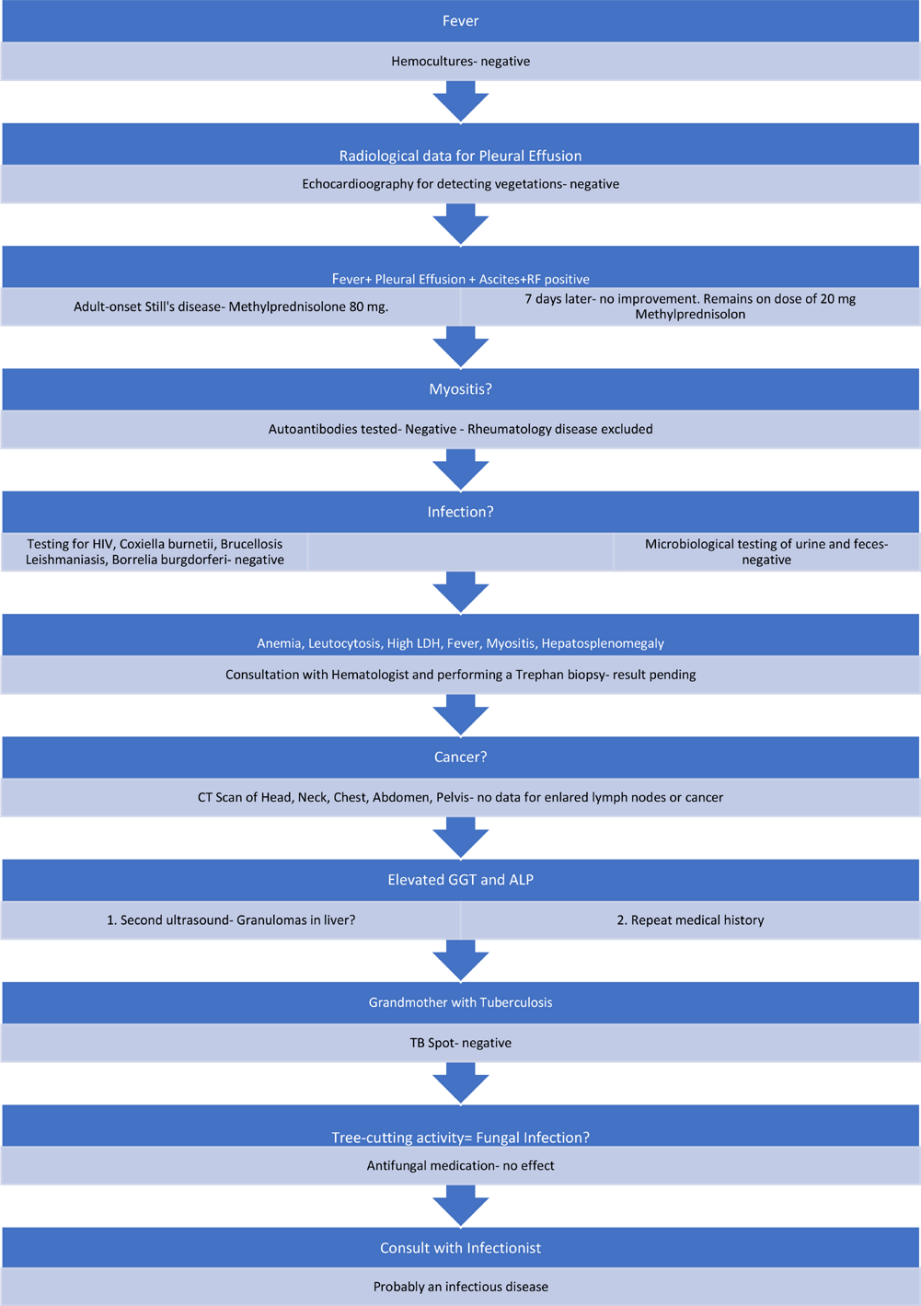
Diagnostic steps in Department of Gastroenterology from October to November.

**Figure 7.**
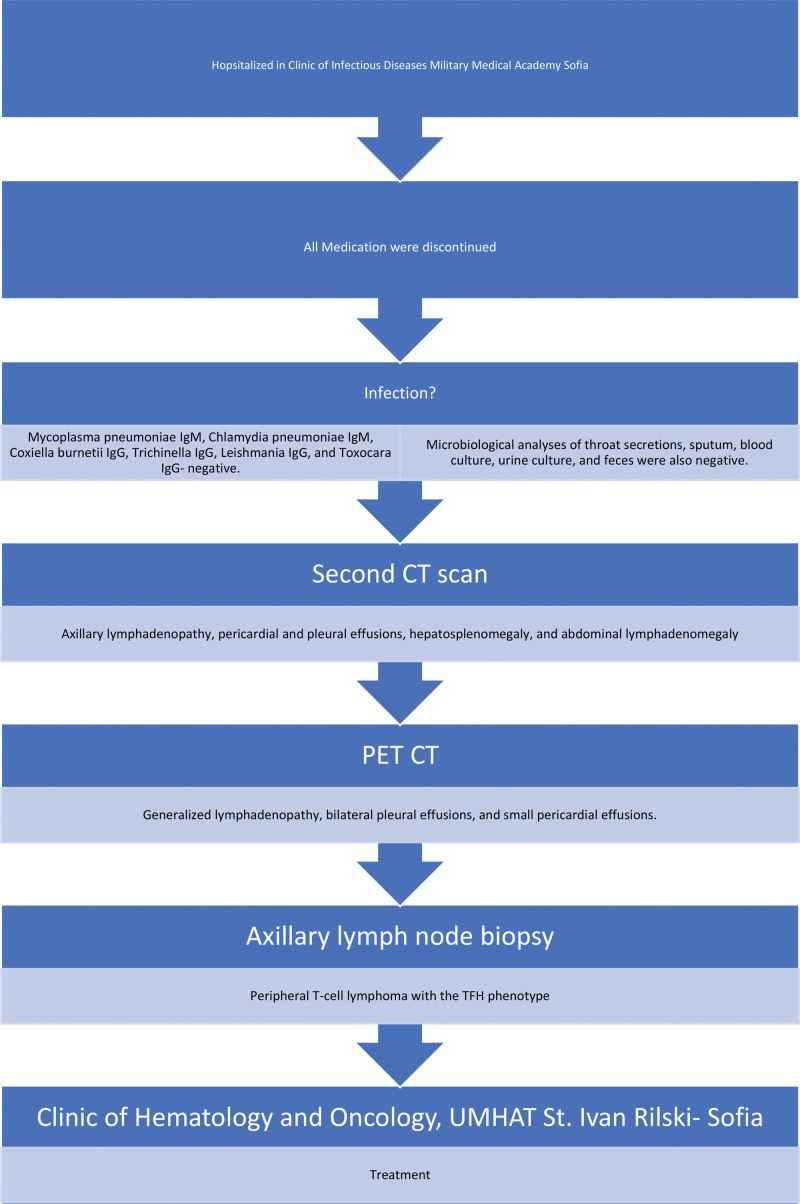
Diagnostic steps in Departments of Infectious Disease Sofia from November to December.

All diagnostic steps are showed in Figures 6 and 7.

The patient has successfully completed 4 rounds of CHOEP with good tolerability as verified by a posttreatment PET-CT scan that indicated a positive response.

## 3. Discussion

The diagnostic process illustrated in this clinical case highlights the critical importance of an exhaustive and repetitive diagnostic approach. Our starting point was the most probable scenario—an infection acquired at home or at work. Repeated inflammation and severe pain in large joints have redirected our attention towards rheumatology numerous times. Sustained treatment for various complications like bacterial infections, candidiasis, disseminated intravascular coagulation, hepatitis B, and replacement therapy permits the progression and evolution of the disease, aiding the diagnostic process through noticeable morphological changes. Thorough surveillance and timely reassessments using advanced imaging techniques such as PET-CT scans, if available, in conjunction with consultations from various specialists and retesting for potential causative agents, can reveal definitive changes that may otherwise go unnoticed.

FUO is defined as a fever with a temperature of 38.3°C (101°F) or higher that persists for at least 3 weeks and remains undiagnosed after an appropriate initial evaluation.^[1]^ When evaluating a patient with FUO, there are several potential etiologies that should be considered: infections (bacterial, viral, fungal, or parasitic infections as well as tuberculosis, endocarditis), autoimmune and inflammatory diseases (systemic lupus erythematosus, rheumatoid arthritis), and malignancy (lymphoma and leukemia). The initial evaluation of a patient with FUO should include a detailed history and physical examination, as well as a thorough laboratory and radiographic workup. This may include complete blood count, urinalysis, chemistry panel, blood cultures, chest X-ray, and other tests based on the clinical suspicion. Additional testing, such as serologic studies, imaging procedures, and biopsy of organs or lesions, may be necessary if the initial workup is negative or inconclusive.

In their retrospective analysis, Zhou et al^[2]^ reported that out of 1641 patients with FUO, 1504 were successfully diagnosed. Among them in 48.69% (799) FUO were due to infectious diseases, with tuberculosis being the most common, accounting for 19.50% (320) of cases. Connective tissue diseases accounted for 19.26% (316) of cases, with adult-onset Still disease being the most common subtype, comprising 89 (5.42%) cases. Neoplastic diseases accounted for 16.94% (278) of cases, with lymphoma (143, 8.71%) being the most common type of cancer. Other diseases were responsible for 6.76% (111 cases) of the cases, and the cause was unclear in 8.35% (137 cases) of the cases.

The 2022 meta-analysis of Wright et al^[3]^ looks at the rates of FUO in different geographic areas and the most common causative agents in those areas. Data from the article are shown in Table 1.

**Table 1:** Incidents and common FUO in different geographic areas.

Geographic region	Incidence of FUO among infectious diseases (in %)	Most common infection
Eastern Mediterranean	Between 33 and 42	1. Brucellosis 2. *Mycobacterium tuberculosis* 3. Infective endocarditis
European	30	1. *Mycobacterium tuberculosis* 2. Infective endocarditis 3. Renal and prostate infection
Northern European countries	14–25	1. Infective endocarditis 2. *Mycobacterium tuberculosis* 3. CMV
South-East Asian Region	44–55	1. *Mycobacterium tuberculosis* 2. Abscesses 3. Brucellosis
Western Pacific Region	56	1. *Mycobacterium tuberculosis* 2. Brucellosis 3. Pneumonia

CMV = Cytomegalovirus infection, FUO = fever of unknown origin.

The authors Bleeker-Rovers et al^[4]^ reported that the percentage of patients with unexplained FUO ranged from 7% to 53%.

Wu et al^[5]^ conducted a study in China and found that B-cell non-Hodgkin lymphoma and T-cell non-Hodgkin lymphoma were the most common lymphoma types observed in patients with FUO. The subcutaneous lymph nodes, bone marrow, and spleen were the most frequently used sites for diagnostic biopsies in lymphoma patients with FUO.

Nodal peripheral T-cell lymphoma with the TFH phenotype is a rare and aggressive subtype of PTCL.^[6]^ These lymphomas represent about 10% to 15% of all non-Hodgkin lymphomas in Western countries but are more common in Asia.

Patients with this subtype of PTCL have high relapse rates and overall lower survival rates compared to other types of non-Hodgkin lymphomas.^[7]^

In a study by de Leval et al,^[8]^ the 5-year overall survival (OS) rate for patients with angioimmunoblastic T-cell lymphoma, a subtype of PTCL with the TFH phenotype, was around 32%. Another study by Federmann et al^[9]^ reported similar results, with a 5-year OS rate of 37% for patients with angioimmunoblastic T-cell lymphoma.

However, the prognosis of nodal PTCL with the TFH phenotype may be improved in certain cases by the use of more aggressive treatment strategies, such as high-dose chemotherapy followed by autologous stem cell transplantation (ASCT). A study by d’Amore et al found that patients with PTCL who underwent ASCT had a significantly better 5-year OS rate (51%) compared to those who did not undergo ASCT (39%).

Nodal peripheral T-cell lymphoma (PTCL) with the TFH phenotype is primarily treated with chemotherapy as the initial intervention. Given the rarity and aggressive nature of this subtype, a well-established optimal treatment strategy remains elusive. CHOP and similar regimens: PTCL is commonly treated with the first-line CHOP regimen, which includes cyclophosphamide, doxorubicin, vincristine, and prednisone. The response rates and survival outcomes of CHOP and related regimens vary among PTCL patients^[10,11]^

Moving forward, we plan to administer an additional 4 courses of the same regimen.

The patient reports significant improvement in his quality of life after the first 4 courses of treatment.

## 4. Conclusion

The clinical case underscores the necessity of a comprehensive and iterative diagnostic approach, incorporating repeated assessments, interdisciplinary consultations, and advanced imaging techniques to accurately identify and address complex medical conditions.

## Author contributions

**Supervision:** Vesselin K. Koltchakov, Raynichka P. Mihaylova-Garnizova, Aleksandar Y. Yordanov, Rosen K. Nikolov, Zahariy A. Krastev.

**Writing – original draft:** Petar I. Trifonov.

**Writing – review & editing:** Vesselin K. Koltchakov, Raynichka P. Mihaylova-Garnizova, Aleksandar Y. Yordanov, Liliya Sokolova, Zahariy A. Krastev.
